# Association between Left Atrial Function and Survival in Systemic Sclerosis

**DOI:** 10.3390/jcdd11100310

**Published:** 2024-10-07

**Authors:** Adrian Giucă, Xavier Galloo, Maria Chiara Meucci, Steele C. Butcher, Bogdan Alexandru Popescu, Ruxandra Jurcuț, Adrian Săftoiu, Ciprian Jurcuț, Laura Groșeanu, Anca Emanuela Mușetescu, Saad Ahmed, Jeska De Vries-Bouwstra, Jeroen J. Bax, Nina Ajmone Marsan

**Affiliations:** 1 Department of Cardiology, Heart Lung Center, Leiden University Medical Center, Albinusdreef 2, 2300 RC Leiden, The Netherlands; adriangiuca17@gmail.com (A.G.); x.galloo@lumc.nl (X.G.); mariachiarameucci@gmail.com (M.C.M.); s.c.butcher@lumc.nl (S.C.B.); j.j.bax@lumc.nl (J.J.B.); 2 “Prof. Dr. C.C. Iliescu” Emergency Institute for Cardiovascular Diseases, Fundeni Street 258, 022328 Bucharest, Romania; bogdan.a.popescu@gmail.com (B.A.P.); rjurcut@gmail.com (R.J.); 3 Department of Internal Medicine, Craiova University of Medicine and Pharmacy, Petru Rares 2, 200349 Craiova, Romania; 4 Department of Cardiology, Vrije Universiteit Brussel (VUB), Universitair Ziekenhuis Brussel (UZ Brussel), Avenue du Laerbeek 101, 1090 Jette, Brussels, Belgium; 5 Department of Cardiovascular Medicine, Fondazione Policlinico Universitario A. Gemelli IRCCS, Via Giuseppe Moscati 31, 00168 Rome, Italy; 6 Department of Cardiology, Royal Perth Hospital, Perth, Western Australia, 197 Wellington Street, Perth, WA 6000, Australia; 7 Faculty of Medicine, “Carol Davila” University of Medicine and Pharmacy, Eroii Sanitari Bvd no. 8, 050474 Bucharest, Romania; adriansaftoiu@gmail.com (A.S.); laura.groseanu@gmail.com (L.G.); 8 Department of Gastroenterology and Hepatology, Elias University Emergency Hospital, Marasti Bvd 17, 011461 Bucharest, Romania; 9 Department of Internal Medicine, “Carol Davila” Central Military Emergency University Hospital, Calea Plevnei 134, 010825 Bucharest, Romania; cjurcut@gmail.com; 10 Department of Rheumatology, “Sf. Maria” Clinical Hospital, Ion Mihalache Bvd 37–39, 011172 Bucharest, Romania; 11 Department of Rheumatology, Craiova University of Medicine and Pharmacy, Petru Rares 2, 200349 Craiova, Romania; anca.musetescu@umfcv.ro; 12 Department of Rheumatology, Leiden University Medical Center, 2333 ZA Leiden, The Netherlands; s.ahmed@lumc.nl (S.A.); j.k.de_vries-bouwstra@lumc.nl (J.D.V.-B.)

**Keywords:** Systemic sclerosis, left atrial reservoir strain

## Abstract

Systemic sclerosis (SSc) is a multisystemic autoimmune disorder in which cardiac involvement is frequent and portends negative prognosis. Left ventricular (LV) diastolic dysfunction is one of the most common cardiac alterations in these patients, and left atrial (LA) reservoir strain (Ɛ_R_) measurement using speckle tracking echocardiography has been proposed as a novel parameter for a better assessment of LV diastolic function. Therefore, the aim of this study was to test the prognostic value of Ɛ_R_ in a large multicenter cohort of SSc patients. In total, 311 SSc patients (54 ± 14 years, 85% female) were included from two different centers. Echocardiography was performed at the time of first visit, including Ɛ_R_ measurement. Over a median follow-up of 132 (interquartile range: 110 to 157) months, 67 (21.5%) patients experienced the outcome of all-cause mortality. Spline curve analysis identified an optimal cut-off value of 30% for Ɛ_R_, and patients with Ɛ_R_ ≤ 30% showed a 10-year cumulative survival rate of 71% as compared to 88% for patients with Ɛ_R_ > 30% (log-rank *p* < 0.001). At the multivariable Cox regression analysis, Ɛ_R_ was independently associated with the endpoint (HR 1.830; 95% confidence interval (CI) 1.031–3.246; *p* = 0.039) together with age (HR 1.071, 95% CI 1.043 to 1.099; *p* < 0.001), sex (female) (HR 0.444, 95% CI 0.229 to 0.861; *p* = 0.016), and diffusing lung capacity for carbon monoxide (HR 0.969 95% CI 0.956 to 0.982; *p* < 0.001). Ɛ_R_ is of independent prognostic value in SSc and might help optimizing risk stratification in these patients.

## 1. Introduction

Systemic sclerosis (SSc) is a rare chronic autoimmune disorder, with multi-organ involvement and relatively frequent cardiac abnormalities, including left ventricle (LV) systolic and diastolic dysfunction, myocardial fibrosis, as well as conduction and rhythm disturbances, which are associated with adverse clinical outcomes [1].

Particularly, LV diastolic dysfunction has been shown to be an important prognosticator in patients with SSc, but its accurate assessment remains challenging [2]. Current recommendations propose an echocardiographic multi-parametric algorithm, in which maximum left atrium (LA) volume index (LAVi) plays an important role, together with pulmonary pressure estimation, mitral inflow and Tissue Doppler parameters [3]. However, compared to the gold standard of right heart catheterization, this approach still has limited diagnostic accuracy to determine elevated LV filling pressures, and LA strain measurement by speckle tracking echocardiography (STE) has been proposed to improve it. Particularly, LA strain measured during the reservoir phase (Ɛ_R_), reflecting mainly atrial compliance and therefore possibly also atrial fibrosis, has been more recently added to the diagnostic algorithm of LV diastolic dysfunction and was shown to be impaired in different patient populations, preceding LA remodeling and dilation [4,5,6].

Initial studies in SSc patients have shown Ɛ_R_ to be reduced compared to controls even before changes in conventional systolic/diastolic function parameters occurred. Also, studies measuring LA strain with cardiac magnetic resonance imaging suggested a potential prognostic value of this measurement in SSc [7,8,9]. We therefore sought to identify if Ɛ_R_ impairment assessed by echocardiography was associated with all-cause mortality in a large multi-center cohort of SSc patients.

## 2. Materials and Methods

### 2.1. Patient Population

From 2003 until 2019, SSc patients referred for a specifically designed multidisciplinary healthcare program at the Department of Rheumatology of the Leiden University Medical Centre (The Netherlands), and at “Prof. Dr. C. C. Iliescu” Emergency Institute for Cardiovascular Diseases (Bucharest, Romania), and who had a complete comprehensive two-dimensional transthoracic echocardiogram (2DTTE) were included [10]. SSc cases were diagnosed according to the classification system proposed by LeRoy and Medsger [11]. Baseline 2DTTE was selected according to the date of the first visit in the care program of both centers. Exclusion criteria were the following: (1) history of myocardial infarction or severe valvular heart disease; (2) image quality and acquisition not sufficient for LA assessment. Written informed consent was obtained at the time of inclusion in the multidisciplinary healthcare program as was approved by the institutional ethical committee (REU 043). This specific retrospective analysis was approved by the institutional review board of both centers.

### 2.2. Clinical Variables

Disease-related characteristics such as SSc subtype (diffuse or limited), modified Rodnan Skin Score (mRSS), Raynaud’s phenomenon, proximal muscle weakness, synovitis, digital ulcerations, and pitting scars were reported. General medical history, medication use, and cardiovascular risk factors were also noted. Laboratory tests were comprised from N-terminal pro-B natriuretic peptide (NTproBNP), creatine kinase and renal function (assessed by the estimated glomerular filtration rate (eGFR) by the Modification of Diet in Renal Disease formula).

Pulmonary function was assessed by spirometry, including percentage of predicted values for forced vital capacity (FVC), forced expiratory volume in 1 s (FEV1) and diffusing lung capacity for carbon monoxide (DLCO). Presence of pulmonary fibrosis was investigated by high-resolution computed tomography [12,13].

### 2.3. Standard 2DTTE

All patients underwent echocardiography in the left lateral decubitus position using available ultrasound system (Vivid 7, E9 and E95; GE—Vingmed, Milwaukee, Wisconsin) and 3.5—MHz or 5—MHz transducers. Standard M-mode and 2-dimensional, color, pulsed, and continuous wave Doppler images were acquired. A commercially available software—EchoPAC (version 113; GE—Vingmed) was used to digitally store all images for off-line analysis. Images were then analyzed with the dedicated software by an expert reader (AG) with no clinical relationship with the patients.

LV end-diastolic diameter (LVEDD), end-systolic volume (LVESV), ejection fraction (LVEF) and LAVi were measured according to the current guidelines [14]. LV diastolic function was further assessed using multiple parameters: peak early (E) and late (A) diastolic velocities and E-wave deceleration time (DTE), measured on pulsed-wave Doppler recordings of the transmitral flow; E/A ratio; e prime (e’) measured with tissue Doppler imaging (TDI) at the septal and lateral side of the mitral annulus in the apical 4-chamber view; average E/e’ ratio was then calculated [3]. When possible, pulmonary artery systolic pressure (PASP) was estimated by determining the right ventricular (RV) systolic pressure, calculated from the peak gradient of the tricuspid regurgitation, to which the value of the right atrial pressure estimated by inferior vena cava dimensions and respiratory collapse was added. RV systolic function was assessed by measuring the tricuspid annular plane systolic excursion (TAPSE) on M-mode echocardiography [15]. Pericardial effusion was also noted.

### 2.4. Two-Dimensional STE

LV strain was measured in the longitudinal direction, using the 3 standard apical views: 4-chamber, 2-chamber, and 3-chamber. LV global longitudinal strain (GLS) was automatically calculated as the average peak systolic strain of 17 LV segments and expressed as absolute values: the higher the value, the better the myocardial shortening [14].

**Figure 1:**
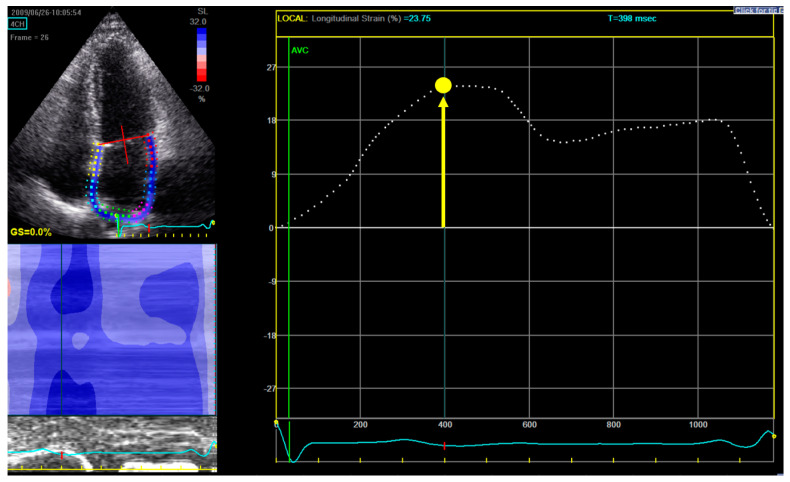
Measurement of left atrial reservoir strain (ε_R_). Upper-left panel shows the selection of the region of interest: in a dedicated non-foreshortened apical 4 chamber view, the tracing begins at the level of the endocardial border of the mitral annulus; it is then continued along the endocardial surface of the left atrium extrapolating across the orifices of the pulmonary veins and left atrial appendage, ending at the opposite side of the mitral annulus. Right panel displays the left atrium longitudinal strain curve: the zero-reference point is at end-diastole and ε_R_ is calculated as the difference between the strain value at the mitral valve opening and ventricular end-diastole (positive value). In this example in a patient from our study, ε_R_ is 23.75%.

For measuring LA longitudinal strain, a dedicated non-foreshortened apical 4-chamber view was used, and the endocardial border tracing was started at the septal part of the mitral annulus, extrapolated across the pulmonary veins and LA appendage and ended at the opposite mitral annulus side (Figure 1) [16].

From the LA strain curve, Ɛ_R_ was measured as the positive peak systolic value within LV end-diastole (mitral valve closure) and mitral valve opening [16]. The zero-reference point was chosen at LV end-diastole (onset of QRS). Ɛ_R_ was selected as the parameter to assess LA function, since it was shown in previous literature to have the best reproducibility and feasibility (also in atrial fibrillation patients) and the strongest prognostic value [17,18,19].

### 2.5. Study Endpoint

An endpoint of all-cause mortality was used. All-cause mortality data were obtained by retrieval of survival status through the municipal civil registries. Time until death was calculated from the initial 2DTTE till date of event.

### 2.6. Statistical Analysis

Categorical variables are expressed as absolute frequencies and percentages. Continuous variables are presented as mean ± SD in case of normal distribution, and as median (interquartile range—IQR) in cases of non-normal distribution. Adherence to normality was assessed by normality tests. Comparison between the groups was performed using the Pearson χ^2^ test for categorical variables and the Mann–Whitney U-test for continuous variables with normal distribution, and Kruskal–Wallis for non-normally distributed continuous variables.

Penalized spline curve analysis was fitted, demonstrating a continuous increase in HR for all-cause mortality across a range of Ɛ_R_ values. To dichotomize the study population for Kaplan–Meier analysis, a cut-off value of 30% was estimated to be the ideal value (i.e., where the predicted HR for all-cause mortality was ≥1).

Kaplan–Meier curves were used to estimate the 10-year survival rates, and differences between the 2 groups (i.e., by using the Ɛ_R_ cut-off) were tested with the Mantel-Cox log-rank test.

To investigate the association between clinical and echocardiographic parameters with the occurrence of the endpoint, univariable Cox Regression was performed. Variables with a univariable value of *p* <0.05 were incorporated into a multivariable model but avoiding collinearity among different variables and overfitting the model based on the number of events. The hazard ratio (HR) and 95% confidence intervals (CI) were reported for each variable. All tests were two-sided and *p* values < 0.05 were considered to be statistically significant. Data analysis was performed using SPSS for Windows, version 25 (IBM Corp., Armonk, New York, NY, USA) and R version 4.0.1 (R Foundation for Statistical Computing, Vienna, Austria).

## 3. Results

### 3.1. Study Population: Clinical and Echocardiographic Characteristics

In total, 375 patients were selected, but 64 patients were further excluded because of insufficient image quality for LA analysis. Therefore, the final analysis included 311 SSc cases, whose clinical and echocardiographic characteristics are summarized in Table 1.

Median time from first non-Raynaud symptom till diagnosis was 3 (IQR 0–17) years, and from Raynaud symptom till diagnosis 2 (IQR 0–10) years. Most cases (70%) were diagnosed as having limited cutaneous SSc (lcSSc) and lung fibrosis was present in 45% of patients.

On average, LV function was preserved as assessed by LVEF (62 ± 6.5%) and LV GLS (median 21%, IQR 19.5–22). LAVi had a median value of 21.8 mL/m^2^ (IQR 17.3–29), and Ɛ_R_ a mean value of 35.7% (± 11.4). Pulmonary pressures were in average within normal range (median PASP 25 mmHg, IQR 21–30 mmHg). Few patients exhibited pericardial effusion (5.5%).

### 3.2. Endpoint Analysis

Over a median follow-up of 132 (IQR 110–157) months, a total of 67 patients (21.5%) experienced the endpoint.

**Figure 2:**
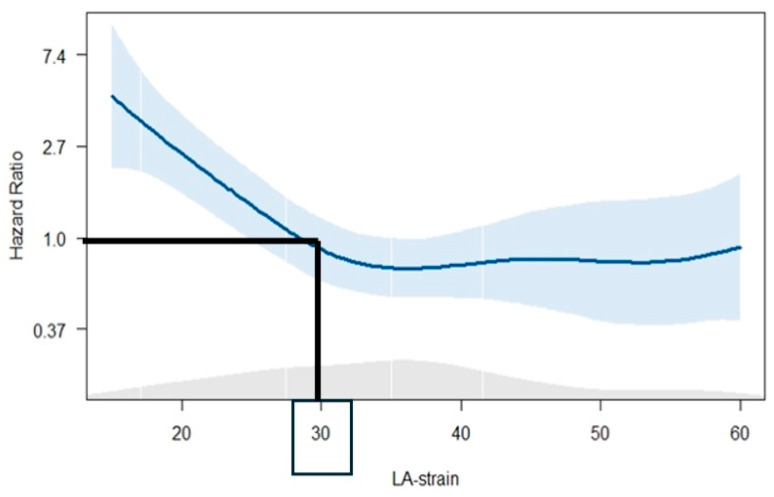
Spline curve demonstrating the HR for all-cause mortality across a range of values of ε_R_.

Spline curve analysis (Figure 2) showed increase in the HR for all-cause death with progressively lower values of Ɛ_R_, and a value of 30% was selected as the optimal cut-off for Ɛ_R_.

**Figure 3:**
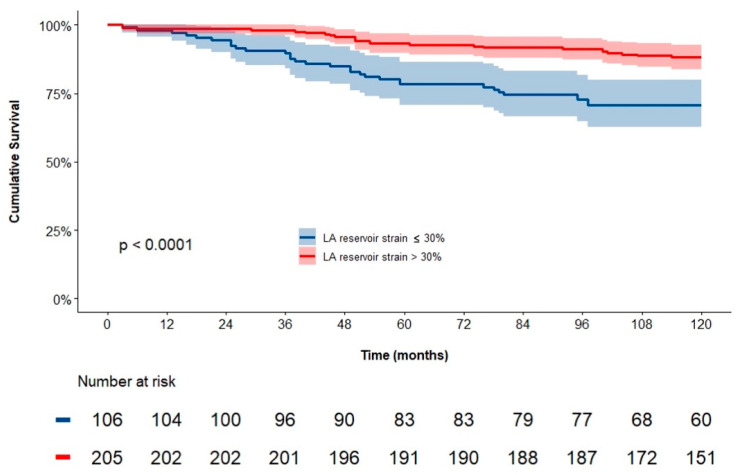
Kaplan–Meier curve for the endpoint of all-cause death at 10 years of follow-up for patients divided according to the cut-off value of ε_R_ of 30%.

Kaplan–Meier analysis demonstrated significantly worse event-free survival for patients with ε_R_ ≤ 30%, when compared to ε_R_ > 30% (Figure 3). In particular, the 10-year cumulative survival rate was 82% for the total population, with 71% and 88% for patients with ε_R_ ≤ 30% and ε_R_ > 30%, respectively (log-rank *p* < 0.001).

According to the univariate Cox regression analysis (Table 2), the following clinical and echocardiographic variables were associated with all-cause death: age, sex, SSc subtype, mRSS, pulmonary fibrosis, NTproBNP, DLCO, LV GLS, LAVi, Ɛ_R_ ≤ 30%, E/e’, and PASP.

On multivariate Cox regression analysis, Ɛ_R_ ≤ 30% was independently associated with the endpoint (HR 1.830, 95% CI 1.031 to 3.246; *p* = 0.039) together with age (HR 1.071, 95% CI 1.043 to 1.099; *p* < 0.001), sex (female) (HR 0.444, 95% CI 0.229 to 0.861; *p* = 0.016), and DLCO (HR 0.969 95% CI 0.956 to 0.982; *p* < 0.001); other variables did not reach statistical significance.

### 3.3. Difference in Clinical and Echocardiographic Variables According to ε_R_

To further characterize the patients according to Ɛ_R_ values, clinical and echocardiographic characteristics were compared between patients with Ɛ_R_ ≤ 30% and >30% (Table 1). In total, 106 (34%) SSc cases presented Ɛ_R_ values ≤ 30%. Patients in this group were older, had a higher prevalence of systemic hypertension and atrial fibrillation, but did not show significant difference in terms of SSc-related characteristics. However, they showed more impaired pulmonary function (namely reduced DLCO) and kidney function and higher values of NTproBNP. By echocardiography, patients with Ɛ_R_ ≤ 30% were also characterized by lower LVEF, lower LV GLS, and larger LAVi; they also showed higher values of E/e’ and lower TAPSE.

## 4. Discussion

The main findings of the current study can be summarized as follows: (1) in a large multicenter cohort, SSc patients with impaired left atrium function, defined as Ɛ_R_ ≤ 30% showed lower survival at 10-year follow-up as compared to patients with Ɛ_R_ > 30%; (2) Ɛ_R_ was independently associated with all-cause mortality.

### 4.1. Cardiac Involvement in SSc Patients

Cardiac involvement in SSc is a challenging diagnosis that, however is an important determinant of adverse prognosis [1,20]. In particular, myocardial dysfunction in these patients is caused by inflammation, ischemia, and fibrosis but it cannot be easily detected [21].

Therefore, even though most patients with SSc initially present without cardiac symptoms, occult primary cardiac involvement may be present and responsible for a significant death rate, reaching 70% at 5 years. As such, careful and timely identification of cardiac involvement is crucial and should include most advanced diagnostic techniques [22,23,24,25].

Systolic dysfunction can be diagnosed early in the course of the disease with more sensitive echocardiographic parameters, such as LV GLS. Van Wijngaarden et al. showed in 234 SSc cases, followed-up for a median period of 2.3 years (IQR 1.3–3.9), that LV GLS reduction occurs in a relative short timeframe from diagnosis, even though LVEF did not change over time [26]. In their study, a significant reduction in GLS (i.e., ≥15%) was found in 19% of cases, and these patients were more likely to have proximal muscle weakness, lung fibrosis, renal impairment and elevated NTproBNP at follow-up, alongside a higher risk of all-cause mortality [26].

However, in SSc patients, LV diastolic dysfunction is four to five times more frequent as compared to systolic dysfunction. Its assessment is based on a multi-parametric algorithm, in which LAVi plays a central role [3,27]. However, LAVi has limited diagnostic value in the early stages of diastolic dysfunction, while changes in left atrial function have been shown to precede LA dilatation and remodeling and to better correlate with LV filling pressures [4,5,28,29]. Therefore, in the latest EACVI consensus document on imaging in heart failure with preserved ejection fraction, Ɛ_R_ is recommended as an additional parameter for the evaluation of LV filling pressures [6].

LA strain and its association with outcome have been evaluated in different studies including different cardiovascular disorders, such as ischemic and valvular heart disease, diabetes, hypertension, and atrial fibrillation [19,30,31,32,33]. Furthermore, Ɛ_R_ showed a negative association with age [34], and atrial fibrosis quantified by late gadolinium enhancement in CMR studies [35,36].

In SSc, Agoston et al. compared 42 cases with 42 age- and gender-matched controls [7]. The two groups did not differ in respect to LV systolic function or LAVi, but patients with SSc had lower values of Ɛ_R_ and higher E/e’ [7]. As such, authors concluded that changes in LA strain are able to mirror myocardial fibrosis before abnormalities in LAVi occur, and suggested to use alterations in LA function as an early indicator of cardiac disease in SSc [7].

Another study comparing 72 SSc cases with 30 healthy controls found two thirds of the SSc patients with LV diastolic dysfunction and with reduced Ɛ_R_ across all SSc subgroups, further emphasizing that the alteration of LA mechanics might represent an early indicator of cardiac disease in SSc [8]. However, no outcomes were reported by these initial studies.

Recently, a multicentric retrospective study that used feature-tracking CMR-derived strain evaluation of the LV and LA analyzed 100 SSc patients for two endpoints: NYHA class II–IV heart failure symptoms at the time of CMR and all-cause mortality at follow-up [37]. Based on spline curve analysis, a cut-off of 27% for Ɛ_R_ was proposed, which was very close to the one identified in the current study [37]. After dichotomizing patients in NYHA I class (39%) and NYHA II-IV class (61%) they observed that Ɛ_R_ was associated with the presence of heart failure symptoms at baseline and with all-cause mortality at follow-up [37]. In addition, Ɛ_R_ demonstrated incremental prognostic value over the variables known to alter the prognosis of SSc patients [37]. Although this study demonstrated for the first time that lower values of LA strain are associated with higher rates of mortality at follow-up, it was performed using CMR, an advanced imaging method that is not readily available and cannot be repeated easily at follow-up. To the best of our knowledge, our study is the first to assess the correlation between Ɛ_R_ measured by echocardiography and all-cause mortality in a large SSc population. By demonstrating its independent association with mortality, it therefore suggests that this parameter might be used to (timely) identify cardiac involvement and, as such, patients at high risk that might merit closer follow-up or specific treatments.

### 4.2. Limitations

A few limitations of the current study should be mentioned. The analyzed population included SSc patients referred for screening and extensive evaluation within a specific healthcare program conducted in tertiary care centers, which could have included selection bias (also in terms of disease characteristics). The measurement of LA strain was performed using one vendor and the cut-off values proposed might be not applicable to other echocardiographic vendors. Treatment strategies and their potential association with the outcome were not considered during follow-up; however, considering the absence of homogeneous recommendations, therapeutic changes were also not included in previously published prognostic models. Overall, the findings of the current study should be confirmed in larger, prospective, multicentric studies.

## 5. Conclusions

In SSc patients, left atrial strain Ɛ_R_ ≤ 30% is independently associated with all-cause mortality, and as such might be useful in the risk stratification of these patients.

## Figures and Tables

**Table 1:** Clinical and echocardiographic characteristics of the study population and comparison between cases with ε_R_ ≤ 30% and ε_R_ > 30%. Values are presented as mean ± SD, median (IQR) or n (%). ACEi—angiotensin-converting enzyme inhibitors; ARB—angiotensin receptor blockers; AZA—azathioprine; CSs—corticosteroids; CYC—cyclophosphamide; dcSSc—diffuse cutaneous SSc; DLCO—diffusing lung capacity for carbon monoxide; eGFR—estimated glomerular filtration rate; FEV1—forced expiratory volume in 1 s; FVC—forced vital capacity; LAVi—left atrial volume index; lcSSc—limited cutaneous SSc; LVEDD—left ventricular end-diastolic diameter; LVEF—left ventricle ejection fraction; LVESV—left ventricular end-systolic volume; LV GLS—left ventricle global longitudinal strain; mRSS—modified Rodnan Skin Score; MTX—methotrexate; NTproBNP—N-terminal fragment of the pro-B natriuretic peptide; PASP—pulmonary artery systolic pressure; RV—right ventricle; SSc—systemic sclerosis; TAPSE—tricuspid annular plane systolic excursion; ε_R_—left atrial reservoir strain.

Variable	Overall Population (n = 311)	ε_R_ ≤ 30% (n = 106)	ε_R_ > 30% (n = 205)	*p*-Value
Age (years)	53 (±14)	61 (±12)	50 (±14)	*<0.001*
Sex (female)	264 (85%)	88 (83%)	176 (86%)	0.508
SSc subtype	174 (70%) lcSSc 76 (30%) dcSSc	62 (70%) lcSSc 26 (30%) dcSSc	112 (69%) lcSSc 50 (31%) dcSSc	0.829
Time from first non-Raynaud’s phenomenon symptom to diagnosis (years)	3 (0–17)	0.5 (−5–20)	3 (0–12)	0.820
Time from onset of Raynaud’s phenomenon to diagnosis (years)	2 (0–10)	2 (0–14)	2 (0–9)	0.948
mRSS	4 (1–6)	4 (2–7)	3 (0–6)	0.118
Raynaud’s phenomenon	301 (97%)	100 (94%)	201 (98%)	0.067
Digital ulcerations	68 (22%)	18 (17%)	50 (24%)	0.129
Pitting scars	125 (40%)	37 (35%)	88 (43%)	0.161
Proximal muscle weakness	27 (9%)	12 (11%)	15 (7.5%)	0.256
Synovitis	35 (12%)	11 (10.5%)	24 (12%)	0.659
Atrial fibrillation	15 (5%)	10 (9.4%)	5 (2.4%)	*0.006*
Hypertension	89 (29%)	45 (42.5%)	44 (22%)	*<0.001*
Smoking	148 (48%)	58 (55%)	90 (44%)	0.076
Diabetes mellitus	13 (4%)	4 (3.8%)	9 (4.4%)	1
Coronary artery disease	13 (4%)	6 (5.7%)	7 (3.4%)	0.353
NTproBNP (pg/mL)	93.8 (53.9–191)	127 (57.6–291)	85 (53–152)	*0.001*
Creatine kinase (U/L)	85 (60–120.7)	84 (58–140)	85 (61–117)	0.982
eGFR (ml/min/1.73 m^2^)	89.4 (±26)	89 (±26)	92 (±26)	*0.009*
DLCO (%pred)	64.2 (±19)	60 (±18)	66 (±19)	*0.012*
FEV1 (%pred)	95 (±19.3)	93 (±19.2)	96 (±19.4)	0.291
FVC (%pred)	101 (±22)	99 (±22.6)	102.3 (±22)	0.317
LVEDD (mm)	47.2 (±5.4)	47.5 (±5.7)	47 (±5)	0.429
LVESV (ml)	30 (24–39)	31 (25–40)	30 (24–39)	0.258
LVEF (%)	62 (±6.5)	60.3 (±7.3)	62.6 (±5.8)	*0.003*
LV GLS (%)	21 (19.5–22)	20.4 (18–21.6)	21 (20–22)	*<0.001*
LAVi (ml/m^2^)	21.8 (17.3–29)	24.2 (19.8–30.8)	20.7 (16–27.3)	*<0.001*
ε_R_ (%)	35.7 (±11.4)	23.9 (±4.2)	41.7 (±9)	*<0.001*
E wave (cm/sec)	78.2 (±18.3)	77 (±22)	78.6 (±16)	0.554
A wave (cm/sec)	71 (58–89)	75 (64–96)	69 (57–83)	*0.009*
E/A ratio	1.04 (0.8–1.3)	0.95 (0.77–1.1)	1.1 (0.89–1.3)	*<0.001*
E/e’ ratio	8.5 (7–10.7)	10 (8–12)	8 (7–10)	*<0.001*
TAPSE (mm)	22.6 (±3.7)	22 (±4)	23 (±3)	*0.013*
Moderate or severe tricuspid regurgitation	18 (17%)	11 (23%)	7 (12%)	0.150
Tricuspid regurgitation maximum gradient (mmHg)	22 (18–27)	24 (18–29)	21.5 (18.3–25)	*0.028*
PASP (mmHg)	25 (21.2–30)	27 (21–30.7)	24.7 (21.3–28.6)	0.190
Pericardial effusion	17 (5.5%)	8 (7.6%)	9 (4.4%)	0.237
Medication				
ACEi or ARB	118 (38%)	51 (48%)	67 (33%)	*0.010*
Beta-blocker	27 (8.7%)	16 (15%)	11 (5.4%)	0.060
CSs	45 (14.5%)	15 (14.2%)	30 (14.7%)	0.520
CYC	7 (2.3%)	1 (1%)	6 (3%)	0.429
MTX	42 (13.5%)	17 (16%)	25 (12%)	0.384
AZA	17 (5.5%)	6 (5.7%)	11 (5.4%)	0.555

**Table 2:** Univariable Cox regression analysis to identify clinical and echocardiographic variables associated with all-cause mortality. HR—hazard ratio; CI—confidence interval; abbreviations as in Table 1.

Variable	Univariable Analysis	
	HR (95% CI)	*p* value
Age (years)	1.060 (1.038–1.082)	*<0.001*
Sex (female)	0.445 (0.259–0.765)	*0.003*
SSc subtype	1.823 (1.099–3.026)	*0.020*
mRSS	1.048 (1.027–1.069)	*<0.001*
Pulmonary fibrosis	2.750 (1.636–4.621)	*<0.001*
NTproBNP (pg/mL)	1.001 (1.001–1.001)	*<0.001*
Creatine kinase (U/L)	1.000 (0.998–1.002)	0.954
eGFR (mL/min/1.73 m^2^)	0.993 (0.985–1.002)	0.119
DLCO (%pred)	0.963 (0.953–0.974)	*<0.001*
LVEDD (mm)	1.016 (0.972–1.062)	0.478
LVESV (mL)	1.008 (0.991–1.025)	0.383
LVEF (%)	0.975 (0.942–1.010)	0.164
LV GLS (%)	0.771 (0.716–0.830)	*<0.001*
LAVi (mL/m^2^)	1.041 (1.018–1.065)	*<0.001*
ε_R_ (≤ 30%)	2.460 (1.522–3.975)	*<0.001*
E wave (cm/s)	0.999 (0.985–1.013)	0.884
E/e’ ratio	1.070 (1.023–1.119)	*0.003*
TAPSE (mm)	0.960 (0.900–1.023)	0.209
PASP (mmHg)	1.086 (1.061–1.112)	*<0.001*
Pericardial effusion	0.805 (0.253–2.564)	0.714
Hypertension	1.927 (1.178–3.154)	*0.009*
Atrial fibrillation	2.098 (0.906–4.856)	0.084

## Data Availability

The data that support the findings of this study are not openly available due to reasons of sensitivity and are available from the corresponding author upon reasonable request.
